# Assessment of specific risks for the recurrence of deep vein thrombosis: a case report

**DOI:** 10.4076/1757-1626-2-7024

**Published:** 2009-08-24

**Authors:** Joshua A Boys, Christine J Medaugh, Houria I Hassouna

**Affiliations:** Department of Medicine, Division of Thrombosis, College of Human Medicine, Michigan State UniversityB-214 Clinical Center East Lansing, MI 48824USA

## Abstract

**Introduction:**

Venous thromboembolism is a multifactorial disease defined by multiple interactions between genetic and environmental components. It is managed by oral anticoagulation with warfarin sodium (Coumadin), a drug that targets the vitamin K epoxide reductase to prevent the recycling of vitamin K epoxide to the reduced form of vitamin K. The reduced form of vitamin K is an essential cofactor in the formation of active clotting factors II, VII, IX, X and regulatory factors protein C, and cofactor protein S through gamma-glutamyl carboxylation. The duration of Coumadin treatment, three to six months or life-long, should be based on the individual risk for recurrent deep vein thrombosis and on the associated increased risk for bleeding complications.

**Case Presentation:**

A previously healthy 50-year-old white male developed a deep vein thrombosis consequent to surgical placement of a titanium rod to correct a fracture of the femur and he was maintained for over a year on daily oral doses of Coumadin 9 mg and aspirin 325 mg. When he began to bruise spontaneously with multiple large hematomas appearing without provocation, he requested that his primary care physician reconsider the anticoagulation. Because of his age, sex, and the possibility of an inherited or acquired anticoagulant protein deficiency he was maintained on Coumadin and a thrombophilia work up was ordered. Test results were interpreted as deficiencies in both protein C and protein S and he was instructed that life-long therapy with Coumadin was necessary. Is this a correct evaluation by his primary care physician?

**Conclusion:**

This case illustrates that Coumadin, a vitamin K agonist, was exerting a therapeutically acceptable negative influence on plasma activity levels of vitamin K-dependent protein C and protein S. Relying on the outcome of a thrombophilia work-up for a decision to maintain or cease Coumadin treatment of patients at risk for recurrent deep vein thrombosis has pitfalls that can be avoided. The use of real-time B-mode venous ultrasonography to verify complete restoration of venous flow before ceasing Coumadin treatment is not always considered in the long-term management of a patient with a first thrombosis, despite the well documented significant risk of deep vein thrombosis recurrence associated with an unresolved thrombosis.

## Introduction

In the mid 20^th^ century, Dr Paul Link noted that cows eating spoiled clover died of hemorrhage. The substance in spoiled clover was purified and was found to be a racemic mixture (R and S isomers) of the anticoagulant drug (3-(α-acetonylbenzyl)-4-hydroxycoumarin) warfarin. The action of warfarin S isomer on VKORC1 prevents vitamin K1 from participating in a post-translational reaction to carboxylate glutamate residues on inactive vitamin K-dependent coagulation factors (factors II, VII, IX, X, and protein C and protein S). This carboxylation reaction is necessary to produce active vitamin K-dependent factors. Warfarin R and S isomers are metabolized in the liver through distinct pathways. The rate of oxidative metabolism of the more active warfarin S isomer is affected by the cytochrome P450 CYP2C9 hydrolase activity, [1] and polymorphisms in the genes encoding this enzyme influence vitamin K1 levels and the degree of blood fluidity in individuals anticoagulated with warfarin. In addition to genetic factors, diet, drugs, and various disease states influence the agonist effect of warfarin on vitamin K. The anticoagulant activity of warfarin sodium (Coumadin) is dependent on absorption from the gut, with levels in the blood peaking about 90 minutes after drug administration, and a plasma half-life of 36 to 42 hours. Over 97% of circulating Coumadin is bound to albumin, with only the small fraction of unbound Coumadin that is biologically active. Coumadin does not act by directly dissolving thrombi, but by targeting the vitamin K epoxide reductase (VKORC1), Coumadin reduces blood clottability, stops the expansion and growth of existing thrombi, prevents formation of new thrombi and allows time for the body’s normal thrombolysis mechanism to resolve existing thrombi. In the absence of vitamin K1 there is a substantial decrease of active vitamin K- dependent factors in blood, and a high risk for bleeding (factors II, VII, IX, X) and thrombosis (protein C, protein S) [2]. Protein C and protein S deficiencies are listed under “thrombophilia”, a term applied to acquired and inherited abnormalities with increased risk for thrombosis. Protein C is the vitamin K-dependent precursor of an anticoagulant enzyme activated protein C (APC). APC aided by protein S markedly decreases the velocity of prothrombin conversion to thrombin by degrading the procoagulant activities of factors V and VIII. Coumadin administration causes decreased protein C activity and a variably normal to decreased antigen protein C concentration, with results that can be misinterpreted as a protein C thrombophilia. If protein C must be assessed while a patient is on Coumadin therapy, levels of protein C activity must be compared to that of prothrombin, factors VII, IX and X and a discrepancy indicating lower protein C activity than expected, strongly suggests a protein C deficiency [3].

Risks of thrombosis and the risk of recurrence of thrombosis differ among the coagulation defects associated with inherited thrombophilia. Higher risks for thrombosis were identified for subjects with antithrombin (risk ratio 8.1, 95% confidence interval [CI], 3.4 to 19.6), protein C (7.3, 95% CI, 2.9 to 18.4) or protein S deficiency (8.5, 95% CI, 3. 5 to 20.8), and factor V Leiden (2.2, 95% CI, 1.1 to 4.7) than for individuals with normal coagulation [4]. The most frequent venous thrombotic manifestation was deep vein thrombosis with or without pulmonary embolism (90% in antithrombin, 88% in protein C, 100% in protein S deficiency, and 57% in factor V Leiden), but a relatively mild manifestation such as superficial vein thrombosis was common in factor V Leiden (43%). The thrombosis risk is compounded by other heterozygous mutations or in association with a chronic disorder, with pregnancy, surgery or trauma. Deficiencies of both protein C and protein S make up 2.5-5% of all persons with initial deep vein thrombosis (DVT) and individuals with protein C deficiency have a 6.5-increased relative risk of initial DVT. Patients with heterozygous protein C deficiency have an incidence of spontaneous thrombosis of approximately 0.4-2.5% per year. Approximately 50-70% of thromboses are spontaneous. By the fifth decade, approximately 50% of patients with heterozygous protein C and protein S deficiency have experienced a thromboembolic event.

## Case presentation

In July 2008, a 50 year-old white male was referred to us for evaluation of the decision by his primary care physician to maintain him on life-long anticoagulation, a decision based on the findings of deficiencies of both protein C and protein S. He is in good health and has no known family history of DVT or coagulation abnormalities. In June 2006, he fell from a tree at work, and a fracture of his right femur was redressed by insertion of a titanium rod. One week after surgery, he complained of pain in his right calf. DVT was confirmed by Doppler ultrasonography and he was anticoagulated with heparin and Coumadin and maintained on 9 mg Coumadin and 325 mg aspirin until June 2007 when he began noticing spontaneous bruising and large hematoma appearing without provocation. He requested that his primary care physician re-evaluate the basis for his anticoagulation. Blood drawn while he was taking a therapeutic Coumadin dose was sent to a regional laboratory for a thrombophilia work up. Results of laboratory tests were significant for protein S activity levels 36% [normal range 54-130%] and protein C activity levels 10% [normal range 70-130%]. Protein C and protein S antigen levels were not performed, and the test results were interpreted as a protein C and protein S deficiency. In our laboratory we drew a blood sample while the patient was taking Coumadin and we prepared citrate platelet poor plasma from blood with the results shown in Table 1. We report a normal coagulation profile and normal response to the prescribed Coumadin dose, with no evidence of thrombophilia. There was no evidence of elevated fibrinogen levels or abnormal fibrinogen variants. Antithrombin levels were 100% as would be anticipated in a patient on Coumadin for such a long period of time. Protein C and protein S were within range expected for the degree of anticoagulation. He was returned to his primary care physician with recommendation that, although he did not have a protein C or protein S deficiency he should remain on Coumadin until resolution of the venous obstruction that occurred consequent to the surgery is confirmed by Doppler ultrasonography. In a healthy male with a first DVT occurring after surgery or trauma, the calculated risk of DVT recurrence is 0.27 very low [5]. It is estimated that 10% of recurrences occur at 2 years and 23% at 5 years post anticoagulation therapy [6]. Unresolved DVT may lead to post-phlebitic syndrome and to a 2.6% annual, 12.4% at 5 years and 16.5% at 7 years increased risk of recurrent DVT [7].

**Table 1.** Results of coagulation studies

Test	Result	Normal Range	Control
Prothrombin Time (PT)	19.7 s	10-12.8 s	11.4 s
Activated Partial Thromboplastin Time (APTT)	36.5 s	20-30 s	26.4 s
Thrombin Clottable Fibrinogen	2.5 g/L	2-4 g/L	
Heat Precipitable Fibrinogen Measured by Lowry’s Chemical Assay	2.5 g/L	2-4 g/L	
Mixing studies with equal volume pooled normal plasma	Corrects PT and APTT		
Variant Fibrinogen	Absent		
Factor II activity	18%^1^	80-100%	
Factor VII activity	9%^1^	80-100%	
Factor X activity	18%^1^	80-100%	
Antithrombin II heparin cofactor activity	100%	70-100%	
Antithrombin III immunoreactive protein	100%	70-100%	
Protein C activity (inactivation of FV & FVIII)	76%^1^	60-100%	
Protein C immunoreactive levels	86%^1^	60-100%	
Protein S immunoreactive free levels	62%^1^	60-100%	

^1^Within expected range for degree of anticoagulation.

Oral anticoagulation was discontinued based on a clotting profile negative for thrombophilia and Doppler ultrasonography pattern consistent with normal blood flow in the deep veins of the right calf and thigh predicting a minimal calculated risk for DVT recurrence.

## Conclusion

It is well established that the duration of Coumadin treatment, three to six months or life-long, should be based on the individual risk for recurrent DVT and on the associated increased risk for bleeding complications [7,8]. Furthermore, knowledge of Coumadin mechanism of action and laboratory monitoring of Coumadin therapy are essential to guide the clinical management of DVT [2]. If protein C must be assessed while a patient is on Coumadin therapy, levels of protein C activity must be compared to those of prothrombin, factors VII, IX and X and a discrepancy indicating lower protein C activity than expected, strongly suggests a protein C deficiency.

Much like thrombophilia, an unresolved DVT has an equally high estimated risk for recurrence of DVT. In a review article published in 2008 by Agnelli and colleagues, the authors cite that a number of studies identify male gender as a risk factor for recurrent DVT [9], a finding first reported in a prospective observational study of 826 patients followed for an average of 36 months. Venous thromboembolism (VTE) recurred in 33 out of 273 men (20%) in comparison with 28 out of 453 women (6%) (RR 3.6. 95% CI 2.3-5.5). Furthermore, they report that residual thrombosis has also been investigated as a risk factor for recurrent VTE. A prospective cohort study was performed to determine the risk for recurrent VTE in patients who have persistent residual thrombosis compared with patients who have early vein re-canalization at the end of a conventional oral anticoagulant treatment. The cumulative incidence of normal results at Doppler ultrasonography was 38.8% at 6 months, 58.1% at 12 months, 69.3% at 24 months, and 73.8% at 36 months. The hazard ratio for recurrent thromboembolism was 2.4 (95% CI, 1.3-4.4; *P = 0.004*) for patients with persistent residual thrombosis versus those with vein re-canalization. The authors conclude that residual venous thrombosis is a risk factor for recurrent VTE. This is the basis for recommending Doppler ultrasonography to rule out the potential of an unresolved DVT [9]. DVT (intravascular obstruction of blood flow) is suspected but cannot be confirmed nor diagnosed by clinical symptoms. The diagnosis of DVT is not by laboratory testing; laboratory testing provides an estimate of risks for DVT recurrence and laboratory testing guides the duration of anticoagulant management. Diagnosis of DVT is obtained exclusively by imaging [10].

## Abbreviations

APC: activated protein C
DVT: deep vein thromboembolism
VKORC1: vitamin K epoxide reductase
VTE: venous thromboembolism
